# Reduction and pH dual-sensitive nanovesicles co-delivering doxorubicin and gefitinib for effective tumor therapy†

**DOI:** 10.1039/c7ra12620d

**Published:** 2018-01-09

**Authors:** Yangui Chen, Xiaoxia Li, Hong Xiao, Jinpeng Xiao, Bo Li, Xiaoyan Chen, Yong Wang, Du Cheng, Xintao Shuai

**Affiliations:** PCFM Lab of Ministry of Education, School of Materials Science and Engineering, Sun Yat-Sen University Guangzhou 510275 China shuaixt@mail.sysu.edu.cn +86-20-84112245 +86-20-84110365; HEC Pharma Co., Ltd. Dongguan 523871 China

## Abstract

Owing to the complexity of tumorgenesis, combination therapy has proven to be a viable strategy for cancer treatment in recent years. However, the delivery and site-specific release of different therapeutic agents remain a major challenge in combination therapy. In this study, a polymeric nanovesicle based on a copolymer of polyethylene glycol and a polypeptide derivative was introduced as a vector to simultaneously deliver hydrophobic gefitinib and hydrophilic doxorubicin hydrochloride for multi-target combination therapy. The vesicle incorporating the two drugs exhibited prominent pH/reduction sensitivities to trigger the release of gefitinib and doxorubicin inside cancer cells. The two drugs co-delivered by the polymeric nanovesicle exhibited a joint anticancer effect both *in vitro* and *in vivo*. In particular, a remarkable therapeutic effect was demonstrated in animal studies using a mouse N2a neuroblastoma model. This study reveals the potential of reduction and pH dual-responsive nanovesicles bearing gefitinib and doxorubicin as an effective nano-medicine for cancer treatment.

## Abstract 2

pH and reduction dual-sensitive polymeric nanovesicles were developed to simultaneously deliver hydrophobic gefitinib and hydrophilic doxorubicin for cancer therapy.

## Graphical abstract

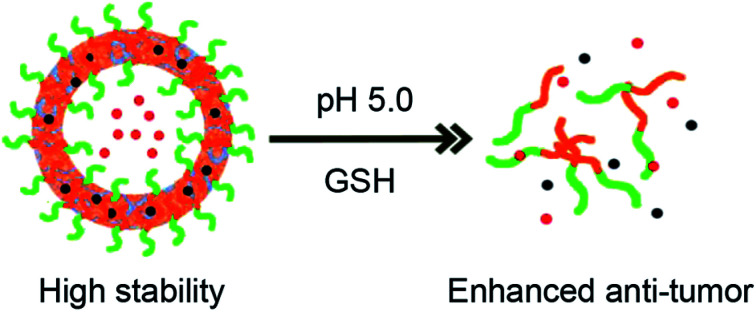

## 1. Introduction

Cancer is one of the most fatal diseases with the second highest mortality after cardiovascular and cerebrovascular disease all around the world.^1–3^ Although tremendous progress has been made recently, developing an effective cancer therapy remains a huge challenge. Among the three main methods employed to treat cancer in the clinic,^4^ surgery and radiation therapies are only suitable for localized cancer, while chemotherapy is able to treat widespread cancer, *e.g.* the metastatic ones. Therefore, chemotherapy alone or in conjunction with radiation therapy or surgery provides a very important option in treating various types of cancer. Unfortunately, the superiority of chemotherapy is affected by the concomitant adverse effects due to non-specific drug distribution and overdosage.^5^ In order to reduce the side effects and overcome drug resistance, combination therapies are usually employed.^6^ In this way, multiple drugs may work synergistically on different but inter-related oncogenic signal transduction pathways and essentially improve the therapeutic outcome.^7^ Among various anticancer drugs, the water soluble doxorubicin hydrochloride (DOX·HCl) is a potent chemotherapeutic agent which inhibits malignant proliferation of cancer cells by interfering with the synthesis of DNA and RNA.^8^ It has been approved for the treatment of many cancers including ovarian cancer, Kaposi sarcoma, breast cancer, and multiple myeloma.^8,9^ On the other hand, gefitinib is a poorly soluble tyrosine kinase inhibitor. It is relatively cytotoxic and has antitumor activity by inhibiting tumor angiogenesis.^10^ To date, some studies have confirmed the therapeutic synergy in combination therapy using tyrosine kinase inhibitor and doxorubicin.^10,11^

Although application of multiple drugs in a cancer treatment *via* common approaches may reduce multi-drug resistance and increase toxicity,^12^ nanomedicine-enabled combination therapy possesses the advantage of less side effects and even less chemoresistance *via* tumor active and passive targeting.^13–15^ Combination therapies based on nano-scale delivery systems are reported to improve anticancer efficacy of two or more drugs mainly through synergistic effects.^12,13^ Among various nano-carriers for drug delivery, nanovesicles assembled from amphiphilic block copolymer represent a unique carrier type capable of simultaneously transporting hydrophilic and hydrophobic drugs due to their aqueous core and hydrophobic membrane compartment.^16–19^ Recently, polymeric vesicles have shown great application potential in cancer chemotherapy and gene therapy.^20^ Yet, tumor tissue or cell-specific drug release remains a big challenge in developing polymeric vesicles-based nano-medicine for cancer therapy.^21,22^

Solid tumor usually exhibits an interstitial microenvironment with low pH (≈6.8), and lysosomal compartments of cancer cells have even lower pH (≈5.0). In addition, cancer cells usually possess high intracellular glutathione (GSH) levels up to millimolar range.^23,24^ The low pH and reducing conditions can be utilized to construct multi-stimulation sensitive nanocarriers to achieve tumor-specific drug release. For instances, pH and reduction dual-sensitive nanocarriers have been prepared for intracellular delivery of small molecular anticancer drugs and protein.^25–27^ In these studies, the stability of nano-medicines *in vivo* was enhanced by formation of disulfide bonds. In addition, our group has developed several reduction and pH dual-responsive micelles based on the block copolymers of poly(ethylene glycol) (PEG) and biodegradable poly(amino acid) derivatives to transport hydrophobic anticancer drugs or siRNA.^28–30^ Because micelles with a solid core were unable to encapsulate hydrophilic drugs under normal conditions, their applications were limited in combination therapy.

**Fig. 1:**
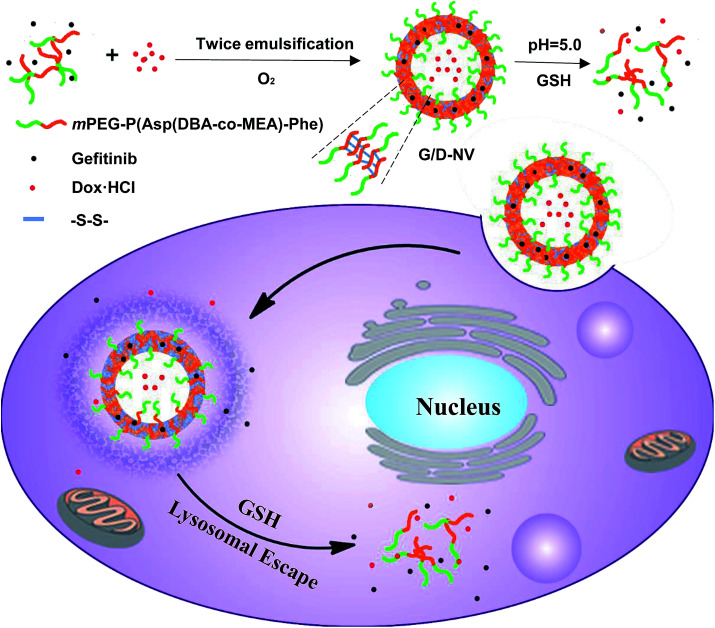
Illustrative preparation of nanovesicle as well as the dual sensitive release of DOX and gefitinib inside tumor cell.

In the present study, an amphiphilic block copolymer of monomethoxyl poly(ethylene glycol) and poly(aspartyl(dibutylethyl-enediamine-*co*-cysteamine)-phenylalanine) (mPEG-P(Asp(DBA-*co*-MEA)-Phe)) was synthesized and employed to prepare a pH and reducing condition dual-responsive nanovesicle for co-delivering hydrophobic gefitinib and hydrophilic doxorubicin hydrochloride into cancer cells. The P(Asp(DBA-*co*-MEA)-Phe) block of copolymer consists of multiple distinct functionalities so that it can form stable vesicle membrane at neutral conditions. In other words, the poly(aspartyl(dibutylethylenediamine)) moiety renders the block pH responsive, hydrophobic at pH 7.4 and hydrophilic at acidic condition, due to the de-protonation–protonation transition of amino groups. In addition, the poly(aspartyl(cysteamine)) moiety allows the carrier to be cross-linked by forming disulfide bonds in oxidizing condition. Furthermore, the introduction of polyphenylalanine can increase the hydrophobicity of the block other than hydrophilic mPEG, which assists the formation of vesicle encapsulating hydrophilic DOX·HCl in the aqueous core and hydrophobic gefitinib in the membrane (Fig. 1). *In vitro* and *in vivo* experiments were carried out to verify the reduction and pH dual-sensitive intracellular drug release of nanovesicle in cancer cells and to explore the potential of this new nanomedicinal system for combination cancer therapy.

## 2. Experimental section

### 2.1 Materials

Methoxy-ε-amino poly(ethylene glycol) (mPEG-OH, *M*_n_ = 2000 Da), anhydrous *N*,*N*-dimethylformamide (DMF) and anhydrous dimethylsulfoxide (DMSO) were purchased from Sigma-Aldrich. Cysteamine, dibutyl ethylenediamine (DBA) and glutathione (GSH) were purchased from TCI, Japan. Triphosgene, *p*-toluenesulfonyl chloride (TsCl) and doxorubicin hydrochloride (DOX·HCl) were purchased from Aladdin, China. l-Aspartic acid benzyl ester was purchased in Adamas, China. l-Phenylalanine, gefitinib and diacetic acid fluorescein (FDA) were purchased from J&K Scientific, China. Dialysis bag (MWCO: 3.5 and 14 kDa) was purchased from Shanghai Green Bird Technology Development Co., Ltd., China. All other reagents were of analytical grade and purchased from Guangzhou Chemical Reagent Factory, China. Ethyl acetate, petroleum ether (60–90 °C), trichloromethane (CHCl_3_) and dichloromethane were dried over CaH_2_ and then distilled under ambient pressure. mPEG-OH was converted into α-methoxy-ε-amino poly(ethylene glycol) (mPEG-NH_2_) as previously described.^31^*N*-Carboxyanhydride of β-benzyl-l-aspartate (BLA-NCA) and *N*-carboxyanhydride of l-phenylalanine (Phe-NCA) were synthesized according to the literature.^28^ DMEM high glucose medium, fetal bovine serum (FBS), double antibiotic (streptomycin/penicillin), phosphate buffer (PBS, pH 7.4), 0.25% trypsin were purchased from Gibco Co., Ltd., China. The MTT test reagents and DAPI were purchased from Sigma-Aldrich, USA. Paraformaldehyde was purchased from Nanjing KeyGen BIOTECH, China. Flow cytometry reagents were purchased from Roche, Switzerland. H&E staining reagents were purchased from Shanghai Hongzi Industrial, China. TUNEL Apoptosis Detection Kit (FragEL™ DNA Fragmentation Detection Kit) was purchased from Merck, Germany.

### 2.2 Cell culture

N2a mouse brain neurons cells were cultured in DMEM high glucose medium containing 10% fetal bovine serum and 1% streptomycin/penicillin in a humidified atmosphere (37 °C, 5% CO_2_). When the cell confluence of 80–90% was reached, they were digested and passaged with 0.25% trypsin for subsequent experiments.

### 2.3 Synthesis of mPEG-P(Asp(DBA-*co*-MEA)-Phe) (PADMP)

Methoxy poly(ethylene glycol)-poly(β-benzyl l-aspartate-phenylalanine) (mPEG-P(BLA-Phe)) was firstly synthesized by ring-opening polymerization of BLA-NCA and Phe-NCA with mPEG-NH_2_ as a macroinitiator.^28^ Briefly, 0.2811 g (0.140 mmol) of mPEG-NH_2_ was vacuum-dried at 70 °C for 4 h in a 50 mL of reaction flask, and then dissolved in 40 mL of anhydrous dichloromethane. Subsequently, 0.60 g (3.14 mmol) of phenylalanine anhydride, 3.50 g (14.05 mmol) of benzyloxycarbonyl aspartic anhydride dissolved in 4 mL of anhydrous DMF were added into the above solution under the protection of argon. The reaction was kept stirring at 35 °C for 48 h, then followed by precipitation into excessive anhydrous ethanol. The precipitate was then centrifuged, washed with anhydrous ethanol, diethyl ether, and finally vacuum dried to obtain 3.2 g of mPEG-P(BLA-Phe) (*M*_n_ = 30 kDa, calculated from ^1^H NMR spectrum). Afterwards, mPEG-P(Asp(DBA-*co*-MEA)-Phe) (PADMP) was synthesized by aminolysis of dibutyl ethylenediamine (DBA) and cysteamine (MEA) with mPEG-P(BLA-Phe). In brief, 2.0 g (0.810 mmol) of mPEG-P(BLA-Phe) was dissolved in 25 mL of anhydrous DMSO, then 0.880 mL (4.275 mmol) of DBA was added into the solution and the reaction was stirred at 35 °C for 24 h. Subsequently, 0.2 g (2.597 mmol) of MEA was added into the solution. After the reaction was stirred at 35 °C for another 48 h, the reaction solution was dialyzed against anhydrous methanol using dialysis bag (MWCO: 3.5 kDa) for 3 days and then concentrated by rotary evaporation, precipitated in diethyl ether, centrifuged, washed and finally dried in vacuum to obtain 1.8 g of pale yellow product PADMP (*M*_n_ = 31 kDa, calculated from ^1^H NMR spectrum).

### 2.4 Preparation of PADMP self-assembled nanovesicle (NV) and drug-loaded nanovesicles

The drug-loaded nanovesicles were prepared using double-emulsion solvent evaporation method.^32^ 20 mg of PADMP and 2 mg of gefitinib were dissolved in 2 mL of chloroform, then 0.2 mL of deionized water containing 1.6 mg DOX·HCl was added and dispersed into a homogeneous emulsion under sonication (VCX130, Sonics, USA, 20 kHz, 40% power level). Subsequently, the primary emulsion was emulsified by sonication in 20 mL of deionized water for 2 min to get the secondary emulsion. After chloroform was evaporated by a rotary evaporator, the vesicle solution was then stirred under bubbling of an oxygen flow for 1 h to crosslink the PAsp(MEA) interlayer *via* disulfide formation and then dialyzed against PBS using a dialysis bag (MWCO: 14 kDa). Finally, the solution was concentrated and washed three times using a MILLIPORE centrifugal filter device (MWCO: 100 kDa) to remove free DOX and then filtered through a syringe filter (pore size: 0.45 μm) to eliminate free gefitinib and large aggregates to obtain gefitinib and DOX co-loaded nanovesicle (G/D-NV). The fluorescein diacetate (FDA) and DOX co-loaded nanovesicle (F/D-NV) was prepared by introducing FDA instead of gefitinib. Nanovesicle loaded with gefitinib (G-NV) or DOX (D-NV) only and blank nanovesicle (B-NV) were prepared similarly except that DOX, gefitinib or both of them were not added, respectively.

### 2.5 Characterization of the polymer and nanovesicle

^1^H NMR spectra were obtained using an AVANCE III 400 MHz nuclear magnetic spectrometer and DMSO-*d*_6_ was used as the solvent. FTIR spectral studies were carried out using a Thermo AVATER 330FT-IR spectrometer in the range between 4000 and 500 cm^−1^ with a resolution of 2 cm^−1^. All powder samples were compressed into KBr pellets in the FTIR measurements.

Raman characterization of G/D-NV was recorded using a Nicolet NXR 9650 Fourier Raman spectrometer. Particle size of the vesicle were measured by dynamic light scattering (DLS). Measurements were performed at 25 °C using 90 Plus/BI-MAS instrument (Brookhaven Instruments Corporation, USA). For each sample, the data from three measurements were averaged to obtain the mean ± standard deviation (SD). Transmission electron microscopy (TEM) images were obtained on a JEM-1400 operated at 120 kV. The sample was prepared by drying a drop (10 μL, 0.1 mg mL^−1^) of G/D-NV solution on a copper grid coated with amorphous carbon. After 2 h, a small drop of uranyl acetate solution (1 wt% in water) was added to the copper grid, which was then blotted with a filter paper after 1 min. The grid was finally dried overnight in a desiccator before TEM observation.

### 2.6 Determination of loading content of DOX and gefitinib

The loading content of DOX·HCl, defined as the weight percentage of DOX in the freeze-dried vesicle, was quantified by UV-vis analysis using a UV-vis-NIR Spectrophotometer (UV-3150, Shimadzu, Japan). After 0.5 mL of the vesicle solution was freeze-dried and weighed, 3 mL of chloroform and DMSO (1 : 1, v/v) were added to redissolve the dried sample. Then the absorbance of DOX at 482 nm was measured to determine the DOX concentration in the solution using a pre-established calibration curve. Similarly, the gefitinib loading content was quantified by high performance liquid chromatography (HPLC, agilent 1260). The dried sample was redissolved in a mixture of chloroform and methanol (1 : 1, v/v). The HPLC conditions were as follows: a C18 column (phenomenex Gemini-NX, 4.6 × 150 mm, 5 μm) was eluted with acetonitrile/water (20 mM KH_2_PO_4_) = 50 : 50 (v/v), and the flow rate was 1 mL min^−1^. The detection wavelength was 252 nm.

### 2.7 *In vitro* release of DOX and gefitinib

DOX and gefitinib release behaviors were studied using dialysis at four experimental conditions, pH 7.4 and pH 5.0 buffers, pH 7.4 and pH 5.0 buffers plus 10 mM GSH. 3 mL of G/D-NV samples were adjusted to the aforementioned conditions and then transferred into dialysis bags (MWCO: 14 kDa). The bags were placed into the same buffered solutions (30 mL). Release study was performed at 37 °C in an incubator shaker (ZHWY-200B, Shanghai Zhicheng, China). At predetermined time intervals, portions of the dialysis solution were removed to quantify the released drugs with HPLC analysis. The cumulative amount of released drug percentages were calculated and plotted against time.

### 2.8 Laser confocal positioning detection

As gefitinib did not fluoresce, we used fluorescein diacetate (FDA) as a substitute for gefitinib, and prepared F/D-NV. The absorption of F/D-NV, distribution of DOX and FDA in the cytoplasm of the N2a cells were observed by laser confocal microscopy. Specifically, N2a cells (5 × 10^3^ cells per well) grown overnight in confocal dishes were incubated with F/D-NV for various lengths of time before the cells were washed twice with PBS and immobilized with 4% paraformaldehyde for 10 min. The cells were then washed twice with PBS and permeabilized with 0.1% Triton X-100 for 10 min, stained with DAPI (1 μg mL^−1^ in PBS) and examined by laser confocal microscopy. The excitation wavelengths for DAPI, FDA and DOX were 405 nm, 488 nm and 514 nm, respectively, and the emission wavelengths were 461 nm, 520 nm and 595 nm, respectively.

### 2.9 Flow cytometry analysis

N2a cells were seeded in 6-well plates at a density of 1 × 10^5^ cells per well. Then samples of G-NV, D-NV and G/D-NV were added into the plates and incubated with cells for 24 h. The cells were trypsinized, washed twice with PBS and collected by centrifugation. 5 μL Annexin V-FITC solution and 5 μL DAPI solution were added and incubated at room temperature for 15 min with cells suspension. The samples were subsequently applied to a BECKMAN COULTER Gallios for flow cytometry analysis. Data analysis was carried out with Kaluza analysis software.

### 2.10 Cytotoxicity evaluated by MTT

N2a cells were seeded into 96-well plates at 5 × 10^3^ cells per well and incubated for 24 h before serial dilutions of nanovesicles were added. After 24 h, 100 μL MTT working solution with final concentration of 0.5 μg mL^−1^ (10 μL of 5 μg mL^−1^ MTT solution in each 90 μL fresh medium) was added into each well of culture plate after discarding the original medium. After 4 h incubation at 37 °C, the supernatant was discarded and 100 μL of DMSO was added to dissolve the precipitate. The absorbance at 570 nm was measured with a microplate reader. The cell survival rate was calculated based on the blank control group. Cell viability = [(absorbance value of the experiment well − absorbance value of blank well)/(absorbance value of control well − absorbance value of blank well)] × 100%. All experiments were performed three times.

### 2.11 Ethical approval and animal tumor model

This study was carried out in strict accordance with recommendations in the Guide for the Care and Use of Laboratory Animals of the National Institutes of Health (NIH publication no. 85-23, revised 1996) and was approved by the Institutional Animal Care and Use Committee of the Sun Yat-sen University (Guangzhou, China). The BALB/c nude mice (Male, 4–6 weeks, 19–21 g) were obtained from Laboratory Animal Center of Sun Yat-sen University, Guangzhou, China. 1 × 10^6^ of N2a cells re-suspended in 0.1 mL of PBS were subcutaneously implanted into the BALB/c nude mice. After the volume of tumors reached to 50 mm^3^, *in vivo* studies were conducted on schedule.

### 2.12 *In vivo* fluorescence imaging

Nanovesicle loading 1,1-dioctadecyl-3,3,3,3-tetramethylindotricarbo-cyanine iodide (DiR) and DOX was intravenously injected into the tumor-bearing mice with a DiR dose of 1 mg kg^−1^ body weight. The fluorescence images were captured on a small animal *in vivo* fluorescence imaging system (Carestream, USA) at the predetermined time points. The mice were anesthetized by inhalational anaesthesia of isoflurane.

### 2.13 *In vivo* anti-tumor effect of nanovesicles

The mice were randomly divided (*n* = 6) and injected with 200 μL of PBS, B-NV, D-NV, G-NV, and G/D-NV, respectively *via* the tail vein. The tumor diameter was measured by the vernier caliper every two days and the tumor volume was caculated by the formula: *V* (mm^3^) = 0.5 × *L* × *w*^2^, in which the “*L*” and “*w*” represented the length and width of the tumor. The body weights and tumor volumes were measured every 2 days up to 20 days when a mouse in PBS control group died. At the end of the experiment, tumor tissues were fixed with 4% paraformaldehyde and then subjected to paraffin section processing and H&E staining and TUNEL staining. H&E staining was carried out with hematoxylin–eosin staining kit of KeyGen BIOTECH following the instruction. TUNEL staining was done using an apoptosis detection kit according to the protocol (FragEL™ DNA Fragmentation Detection Kit, Colorimetric-TdT Enzyme). All the sectional samples were observed and taken pictures under an optical microscope. In a separate experiment, five groups of tumor-bearing mice (*n* = 4) were subjected to the same treatment described above to evaluate the animal survival for 34 days.

### 2.14 Statistical analysis

All experimental data were expressed as mean ± standard deviation (mean ± SD), and statistical analysis was done by using the SPSS 19.0 statistical software, and the difference between groups using one-way ANOVA method (one-way ANOVA). *P* < 0.05 indicates a statistically significant difference.

## 3. Results and discussion

### 3.1 Polymer synthesis and characterization

**Fig. 2:**
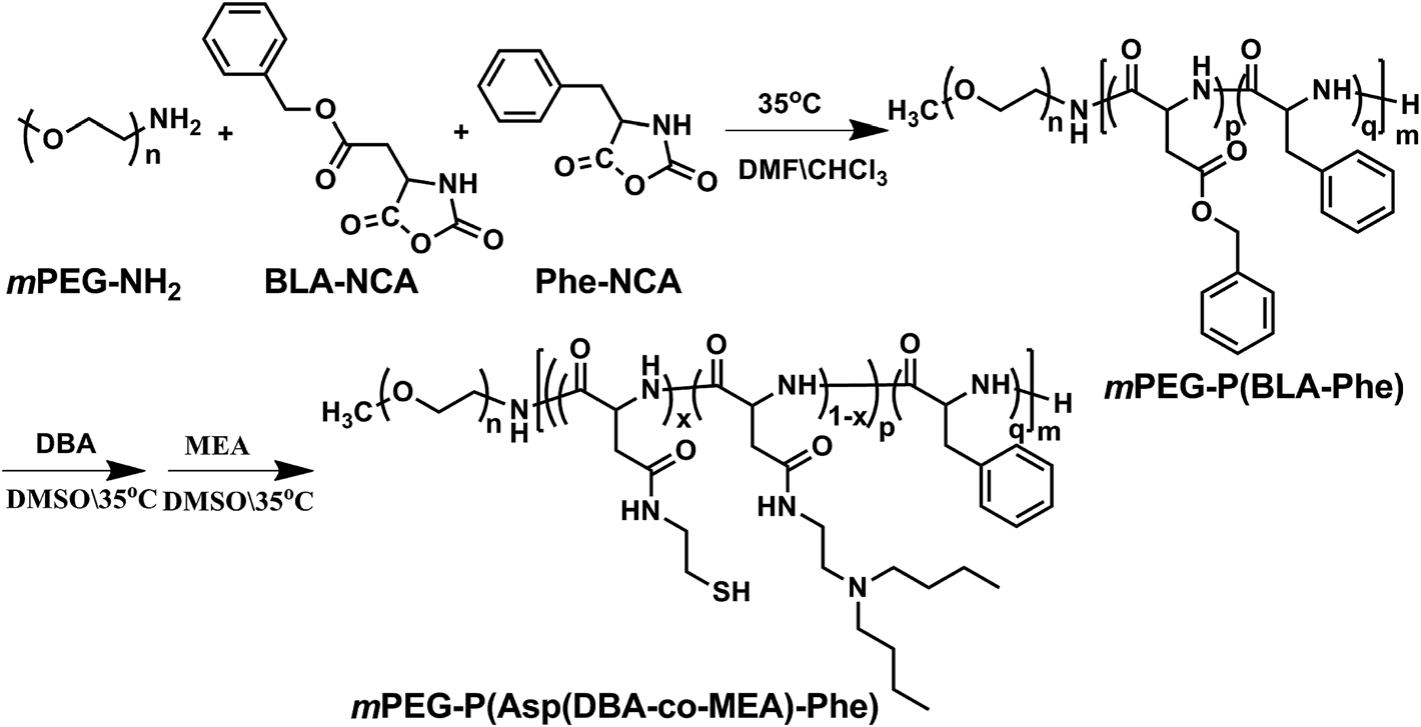
The Synthesis of amphipathic polymer mPEG-P(Asp(DBA-*co*-MEA)-Phe).

The pH and reduction dual responsive copolymer mPEG-P(Asp(DBA-*co*-MEA)-Phe) was synthesized according to a previously reported method (Fig. 2).^31^ Firstly, mPEG-P(BLA-Phe) was synthesized by ring opening polymerization of BLA-NCA and Phe-NCA monomers in DMF/CHCl_2_ using mPEG-NH_2_ as an initiator. Then, this prepolymer underwent aminolysis reaction to remove the β-benzyl protection groups and meanwhile to introduce the tertiary amino-groups and thiol groups, through which the pH and reduction dual responsive copolymer mPEG-P(Asp(DBA-*co*-MEA)-Phe) was obtained.

**Fig. 3:**
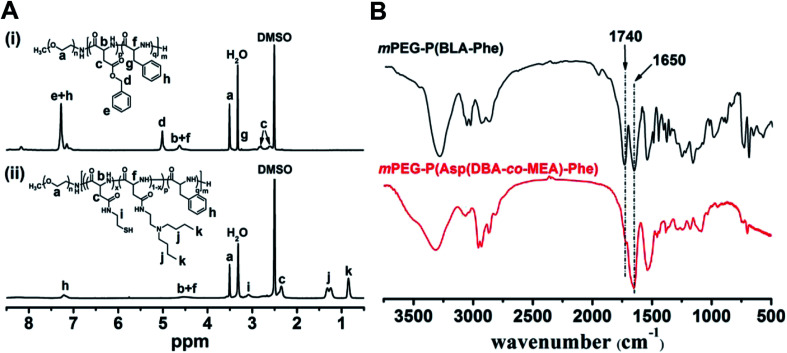
(A) ^1^H-NMR spectrum of mPEG-P(BLA-Phe) (i) and mPEG-P(Asp(DBA-*co*-MEA)-Phe) (ii) in DMSO-*d*_6_. (B) FTIR spectra of mPEG-P(Asp(DBA-*co*-MEA)-Phe) and mPEG-P(BLA-Phe). 1650 cm^−1^ shows *v*_C=O_ of amido bond, 1740 cm^−1^ shows *v*_C=O_ of ester bond.

The ^1^H NMR spectra of mPEG-P(BLA-Phe) and mPEG-P(Asp(DBA-*co*-MEA)-Phe) are shown in Fig. 3A. The assignment of the resonances in the ^1^H NMR spectrum of mPEG-P(BLA-Phe) showed characteristic peaks at 2.5–2.9 ppm (–CHC**H**_**2**_COO–), 3.1 ppm (–C**H**_**2**_C_6_H_5_), 3.5 ppm (–OC**H**_**2**_C**H**_**2**_–), 4.6 ppm (–C**H**CONH–), 5.0 ppm (–COOC**H**_**2**_C_6_H_5_), and 7.1–7.3 ppm (–C_6_**H**_**5**_). NMR analyses also allowed calculation of the repeating units and polymerization degree of the products. It was found that mPEG-P(BLA-Phe) contained on average 116 monomer units by comparing characteristic peak integrals of the methylene moiety (–OC**H**_**2**_C**H**_**2**_–) and the benzene moiety (–C_6_**H**_**5**_). By comparing the integrals of resonance peaks at 3.1 ppm (CH–C**H**_**2**_C_6_H_5_), 5.0 ppm (–COOC**H**_**2**_C_6_H_5_) and 3.5 ppm (–OC**H**_**2**_C**H**_**2**_–), the polymerization degree of BLA and Phe are 97 and 19, respectively. By comparison, it was found that the NMR spectra of mPEG-P(Asp(DBA-*co*-MEA)-Phe) exhibited peaks at 0.85 ppm (–C**H**_**3**_) and 1.15–1.40 ppm (–C**H**_**2**_C**H**_**2**_CH_3_) and no peak appeared at 5.0 ppm (–COOC**H**_**2**_C_6_H_5_), which indicated the successful aminolysis reaction. The FT-IR spectra of mPEG-P(BLA-Phe) and mPEG-P(Asp(DBA-*co*-MEA)-Phe) are shown in Fig. 3B. The characteristic ester band (1730 cm^−1^) disappeared in the spectrum of mPEG-P(Asp(DBA-*co*-MEA)-Phe), showing successful deprotection of β-benzyl groups. These results confirmed that mPEG-P(Asp(DBA-*co*-MEA)-Phe) was successfully synthesized.

### 3.2 Preparation and characterization of nanovesicle

**Fig. 4:**
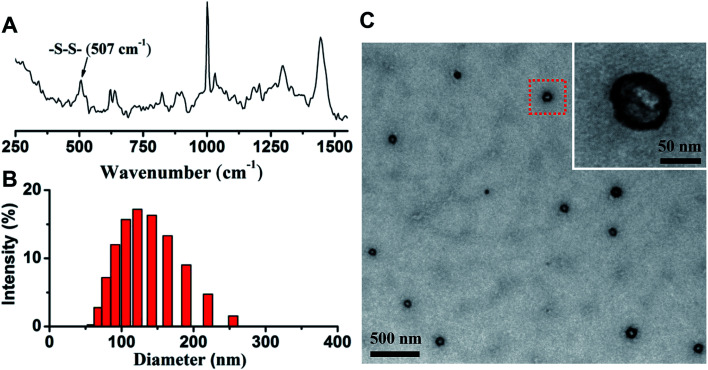
(A) Raman spectrum of the vesicle based on mPEG-P(Asp(DBA-*co*-MEA)-Phe) clearly showing the formation of disulfide bonds. The size distribution (B) and TEM image (C) of G/D-NV demonstrate the formed vesicle structure with diameter around 100 nm.

After the nanovesicle were prepared, Raman spectroscopy was used to determine the formation of disulfide bonds in G/D-NV. As shown in Fig. 4A, the intensive peak at 507 cm^−1^ indicates the formation of disulfide bonds.^33^ Size distribution determined by DLS (Fig. 4B) showed that the particle size of G/D-NV was about 120 nm. TEM analysis (Fig. 4C) demonstrated that the G/D-NV had vesicle structure, spherical morphology, and uniform size around 100 nm. The slight difference in particle size of DLS and TEM measurements was likely due to vesicle shrinkage caused by drying sample for TEM analysis. The loading contents of gefitinib in G-NV and G/D-NV were 1.60% and 1.42%, respectively. The loading contents of DOX in D-NV and G/D-NV were 3.23% and 3.16%, respectively. The encapsulation efficiencies of gefitinib and DOX in G/D-NV were 13.8% and 38.5%, respectively. The percentage of entrapped gefitinib was lower than that of DOX, which could be mainly attributed to their different encapsulation places, *i.e.* hydrophilic DOX was encapsulated in the aqueous core while hydrophobic gefitinib in the membrane.

### 3.3 *In vitro* release of DOX and gefitinib

**Fig. 5:**
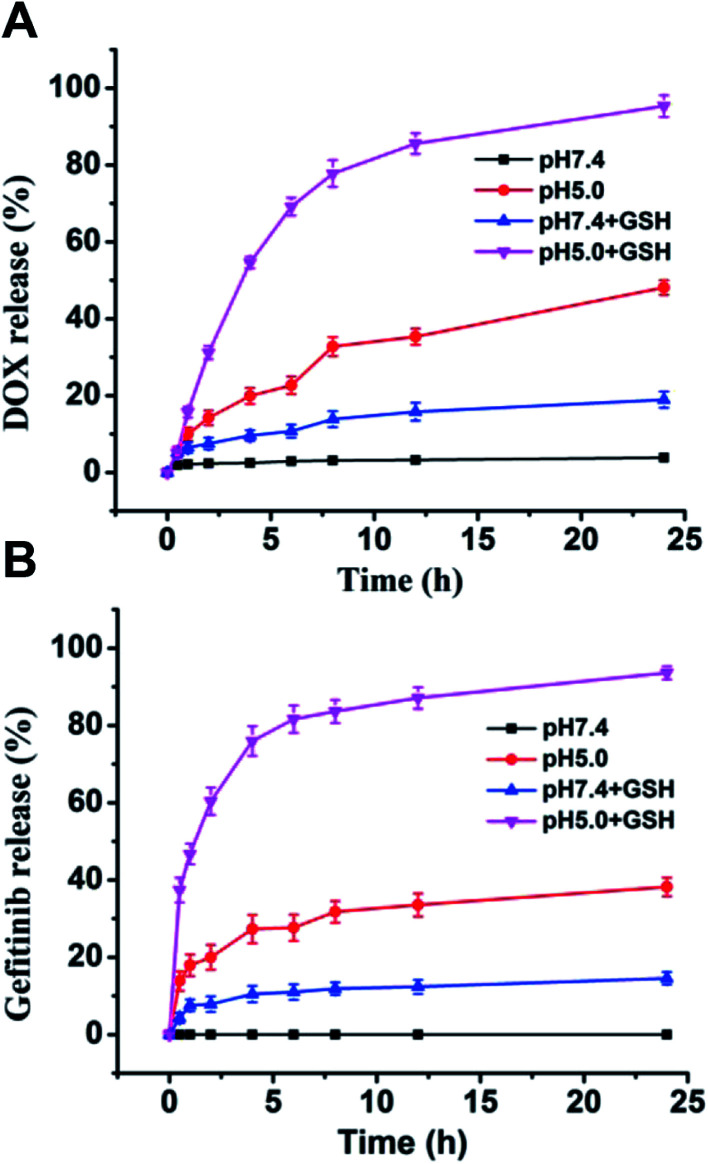
*In vitro* DOX (A) and gefitinib (B) release from G/D-NV at different conditions (GSH concentration 10 mM if added, study performed at 37 °C, data are mean ± standard error of three parallel samples).

The nanovesicle were supposed to release the loaded DOX and gefitinib inside tumor cells by responding to the high GSH concentration and lysosomal low pH.^34^ The release profiles of DOX and gefitinib from vesicle were conducted at different conditions *in vitro*. As shown in Fig. 5, DOX and gefitinib showed analogous release behaviors. Specifically, at pH 7.4 without GSH, there was almost no release for both DOX and gefitinib. After 10 mM GSH was added, more than 10% drug were released in 24 hours for both DOX and gefitinib. The result reveals that the drug leakage ubiquitous for nano-carriers during circulation can be effectively reduced by the introduction of disulfide bond cross-linking. When the pH value of vesicle solution was changed to 5.0, the release of DOX and gefitinib increased to about 40% after 24 hours even without adding GSH, which could be attributed to the protonation of more amino groups causing hydrophilization of hydrophobic capsule PAsp(DBA). Moreover, in the presence of 10 mM GSH, the cumulative release of drug dramatically increased to nearly 80% at pH 5.0 within 6 h. These results indicate that the G/D-NV possess dual sensitivities to allow pH and reduction-triggered drug release inside cancer cells, which can be exploited for tumor site-specific drug delivery.

### 3.4 Cellular uptake and intracellular distribution of nanovesicle

**Fig. 6:**
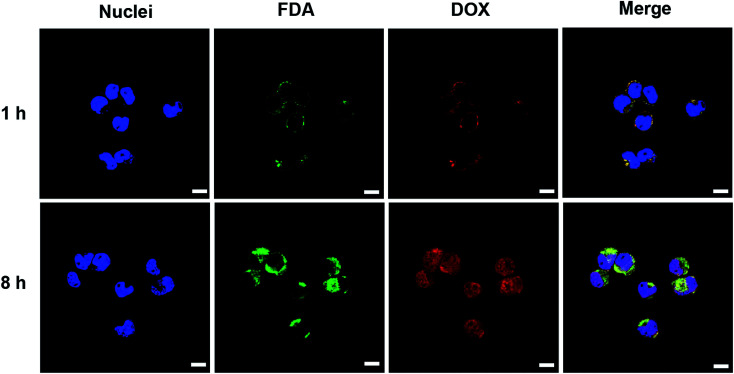
Laser confocal microscopic images of N2a cells after co-incubation with FDA and DOX co-loaded nanovesicle for different time (scale bars: 10 μm). The blue fluorescence is the nuclei stained with DAPI, the green and red fluorescence are the FDA and DOX, respectively.

The cellular uptake, intracellular distribution and intracellular drug release of nanovesicle were studied in N2a cells using laser confocal scanning microscope (LSCM). Hydrophobic fluorescein diacetate (FDA) instead of the non-fluorescent gefitinib was loaded into the vesicle for imaging. After N2a cell incubation with vesicle for 1 h, the DOX (red fluorescence) and FDA (green fluorescence) were primarily located in the cytoplasm or around the cell membrane (Fig. 6). In addition, the bright yellow signal produced by the overlapping of FDA and DOX fluorescence indicated the efficient co-endocytosis of both drugs. When the incubation time increased to 8 h, obviously enhanced fluorescence intensities of DOX and FDA in N2a cells were observed. On the other hand, most of the red fluorescence was localized in nuclei, providing a strong evidence that drug release can be triggered inside cancer cell microenvironment because only the released free DOX can travel to the nuclei. These results indicated that DOX and gefitinib were able to be co-delivered into the N2a cells *via* the nanovesicle-mediated delivery meanwhile be released in response to the acidic (pH 4.5–5.5 in lysosome) and reductive (10 mM GSH) conditions inside tumor cell.^34^

### 3.5 Cytotoxicity and cell apoptosis

**Fig. 7:**
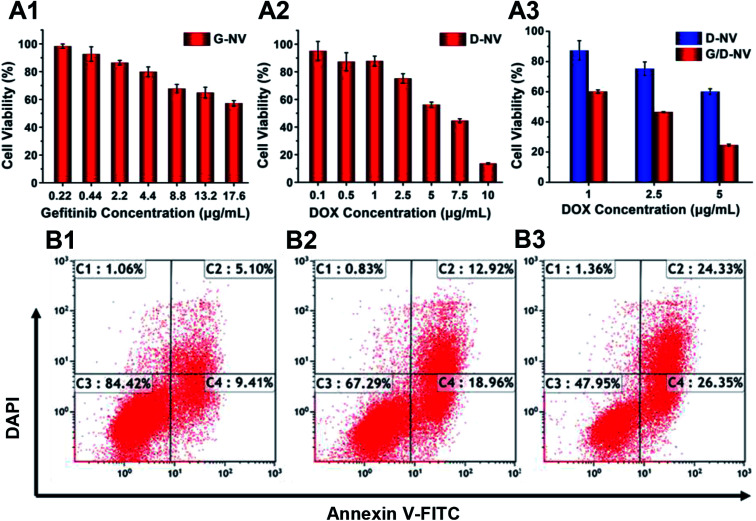
(A1–A3) The cell viability of N2a cells after co-incubation with various nanomedicines ((A1) G-NV, (A2) D-NV, (A3) G/D-NV) evaluated by MTT assay. The gefitinib concentration is 2.24 μg mL^−1^ in (A3). (B1–B3) The apoptotic ratio of N2a cells after incubating with (B1) G-NV, (B2) D-NV and (B3) G/D-NV (gefitinib concentration: 2.24 μg mL^−1^, DOX concentration: 2.5 μg mL^−1^).

The cytotoxicity of blank B-NV, D-NV, G-NV and G/D-NV were evaluated with MTT assay. MTT assays showed that nearly 80% N2a cells incubated with PADEP were viable at a concentration up to 200 μg mL^−1^, indicating PADEP was practically non-cytotoxic (Fig. S1, ESI†). When DOX or gefitinib was loaded into the vesicle, cell viability was decreased obviously with the increase of concentrations of DOX or gefitinib (Fig. 7A1 and A2), indicating that G-NV and D-NV alone can induce apoptosis to certain extent. In addition, compared with single drug treatment of D-NV or G-NV, co-delivery of DOX or gefitinib (G/D-NV) appeared much more potent in killing tumor cells (Fig. 7A3). For example, at the DOX concentration of 1 μg mL^−1^, the presence of gefitinib induced decrease of cell viability from 87.30% to 60.09%. Moreover, the IC_50_ of DOX was decreased from about 7.5 to 2.0 μg mL^−1^. These results imply that the combination of gefitinib and DOX might exert an enhanced anticancer effect.

To further analyze the apoptosis of N2a cells induced by G-NV, D-NV and G/D-NV (DOX concentration: 2.5 μg mL^−1^; gefitinib concentration: 2.24 μg mL^−1^), Annexin V-FITC/PI staining assay was performed and the apoptotic and necrotic cells were quantified by flow cytometry. The percentages of necrotic C1, late apoptotic C2, live cells C3 and early apoptotic C4 are shown in Fig. 7B. The sum of C1, C2 and C4 indicates the ability of vesicles to induce apoptosis in N2a cells. G/D-NV exhibited more potent activity to induce apoptosis (Fig. 7B3), and the apoptosis plus necrosis rate amounted to 52.05%, which was significantly higher than that induced by G-NV group (15.58%) or D-NV group (32.71%). Apparently, DOX and gefitinib acted jointly on cancer cells to induce their apoptosis, which was consistent with the MTT results.

### 3.6 *In vivo* tumor accumulation effect evaluated by fluorescence imaging

**Fig. 8:**
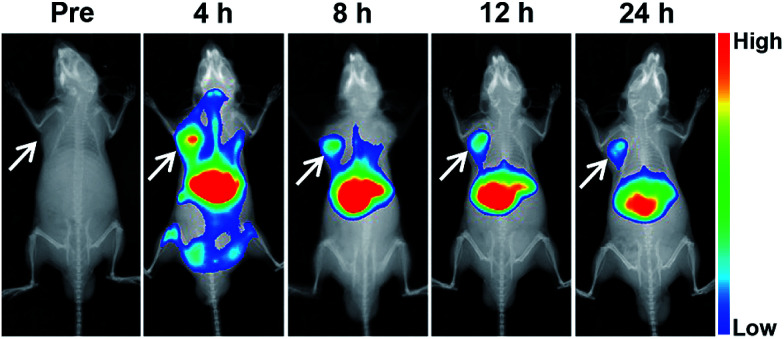
Typical *in vivo* fluorescence images at different times after mouse receiving treatment of DiR/DOX-NV *via* tail vein injection. The arrows point out tumor site. Dose of DiR: 1 mg kg^−1^ body weight.

The tumor accumulation of the nanovesicle was investigated in nude mice bearing mouse neuroblastoma N2a cells xenograft. For *in vivo* fluorescence imaging, near-infrared (NIR) dye DiR instead of gefitinib was loaded into the micelle due to the favorable for clean imaging background of highly tissue-penetrative NIR light.^35^ As shown in Fig. 8, the DiR fluorescence intensity in tumor site reached the highest value at 4 h after injection and maintained stronger than anywhere except liver up to 24 h, indicating the vesicle can accumulated in tumor site effectively. According to the sum fluorescence intensity of the tumor region and the whole body at 4 h after injection, about 5.34% nanoparticle accumulated in tumor site. These results are in line with the previous report that nanomedicines may accumulate preferentially in tumors through the enhanced permeability and retention (EPR) effect and mainly metabolized by liver.^36^

### 3.7 Anti-tumor effect of nanovesicles in mice bearing subcutaneous N2a tumor

**Fig. 9:**
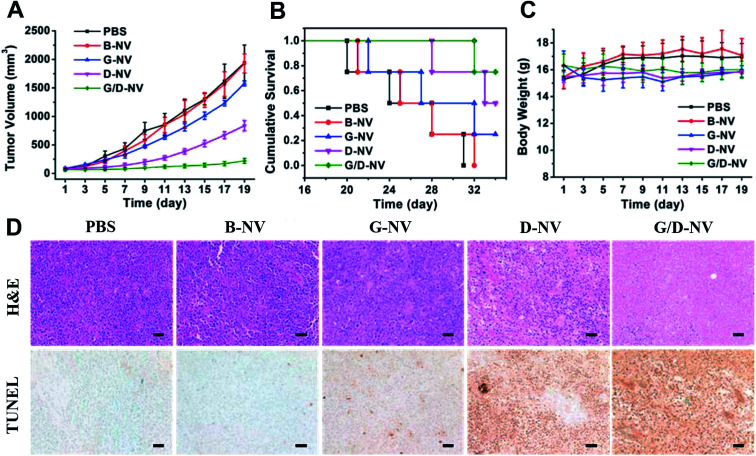
(A) Enhanced tumor growth inhibition of N2a xenografts by G/D-NV (*n* = 6). (B) Cumulative survival of nude mice bearing N2a tumors receiving different treatment (*n* = 4). (C) Body weight of mice receiving different treatments (*n* = 6). Means and standard errors are shown. (D) Representative images of histopathological analysis with H&E and TUNEL staining for each dissected tumor tissue. For TUNEL staining, brown and green stains indicating apoptotic and normal cells, respectively. DOX dosage: 2.5 mg kg^−1^ body weight; gefitinib dosage: 1.12 mg kg^−1^ body weight. Scale bars represent 50 μm.

As the combination of DOX and gefitinib does enhance the cancer cell apoptosis *in vitro* based on the nanovesicles-mediated delivery, whether G/D-NV has significant efficacy in inhibiting tumor growth *in vivo* deserves our attention. The anti-cancer effects of the different drug-loaded nanovesicles were evaluated by the inhibition of tumor growth. As shown in Fig. 9A, the tumors in mice receiving B-NV (1929.3 ± 167 mm^3^) grew to similar size as in the PBS control group (1938.5 ± 312 mm^3^) after 19 days of treatment, indicating the ineffectiveness of blank vesicle in cancer treatment. However, single drug therapies using G-NV and D-NV resulted conspicuous inhibition of tumor growth in mice, showing tumor volumes of 1577.9 ± 43 mm^3^ and 549.5 ± 108 mm^3^, respectively, after 19 days of treatment. Obviously, gefitinib and DOX can be delivered to cancer cells by the nanovesicle *in vivo* and exert therapeutic effect due to the apoptosis-promoting activity of gefitinib and DOX as already proved *in vitro*. In addition, DOX and gefitinib co-loaded nanovesicle (G/D-NV) showed much more potent anticancer efficiency than either G-NV or D-NV. In this case, the tumor volume only grew to 221.4 ± 43 mm^3^ after 19 days of treatment. Consistent with the tumor growth inhibition data, the survival rates of mice were highly dependent on the therapeutic regimens (Fig. 9B). All mice receiving PBS control and B-NV died in 31 and 32 days, respectively. At day 35, the survival rates of mice receiving G-NV and D-NV were 25% and 50%. However, it increased to 75% through the combined treatment strategy with G/D-NV. On the other hand, all the mice receiving the treatment of vesicles showed no significant difference in body weight compared with the control group (Fig. 9C), which implied that the delivery system had minimal systemic toxicity.

The histological changes of tumor after various treatments were examined and compared. As shown in Fig. 9D, H&E stained section of tumor tissue from mice receiving PBS and B-NV both showed obvious cell over proliferation and nuclear polymorphism. By contrast, the hyperplasia of tumor cells was effectively inhibited in tumor tissues for the three therapeutic groups. Especially, the combined treatment of G/D-NV resulted in the fewest tumor cells. In line with the tumor growth inhibition results, mice receiving the treatment of G/D-NV showed the highest level of tumor apoptosis, as verified by the TUNEL staining of tumor sections. All these results evidenced that the combined treatment of DOX and gefitinib based on pH and reduction dual-sensitive nanovesicle delivery system exerted joint anticancer effects *in vivo*.

## 4. Conclusions

In conclusion, an amphiphilic copolymer mPEG-P(Asp(DBA-*co*-MEA)-Phe) was synthesized and demonstrated appropriate for the construction of a nanovesicle to co-deliver the hydrophilic doxorubicin and hydrophobic gefitinib for effective cancer therapy. The nanovesicle showed pH and reduction dual-responsibility, which can be used to effectively deliver anti-cancer drugs into targeting tumor cells. Both *in vitro* and *in vivo* studies proved that the co-delivery of gefitinib and doxorubicin with the nano-carrier had anti-tumor effect far superior to the single drug delivery, which revealed the potential of the reduction and pH dual-responsive nanovesicle as a platform to develop potent nanomedicines for cancer treatment.

## Conflicts of interest

There are no conflicts to declare.

## Supplementary Material

RA-008-C7RA12620D-s001
