# Supplementary material for: Reduction and pH dual-sensitive nanovesicles co-delivering doxorubicin and gefitinib for effective tumor therapy
Source: RSC Adv. 2018 Jan 9;8(4):2082–91. doi: 10.1039/c7ra12620d (PMC9077202; doi:10.1039/c7ra12620d)
Supplement: RA-008-C7RA12620D-s001 [file RA-008-C7RA12620D-s001.pdf]

Electronic Supplementary Material (ESI) for RSC Advances.
This journal is © The Royal Society of Chemistry 2017

Electronic Supplementary Information (ESI)

Reduction and pH dual-sensitive nanovesicle co-delivering Doxorubicin and Gefitinib for effective tumor therapy

Yangui Chen[a], Xiaoxia Li[a], Hong Xiao[a], Jinpeng Xiao[b], Bo Li[a], Xiaoyan Chen[a], Yong Wang[a], Du Cheng[a], Xintao Shuai*[a]

[a]PCFM Lab of Ministry of Education, School of Materials Science and Engineering, Sun Yat-Sen University, Guangzhou 510275, China.

[b]HEC Pharma Co., Ltd., Dongguan 523871, China

*Correspondence should be addressed to:

Xintao Shuai

Tel.: +86-20-84110365

Fax: +86-20-84112245;

E-mail: shuaixt@mail.sysu.edu.cn

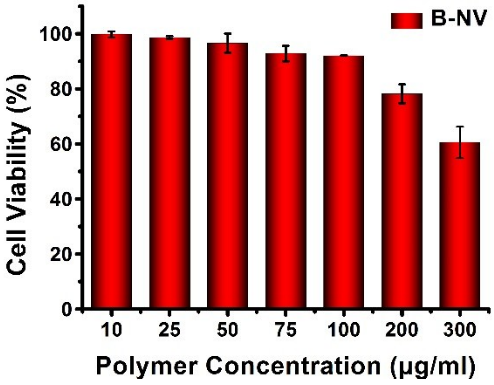

Fig. S1 Cytotoxicity of polymer in N2a cells at different polymer concentrations. Incubation time: 24 h.
